# Systematic Evaluation of the Metabolic to Mitogenic Potency Ratio for B10-Substituted Insulin Analogues

**DOI:** 10.1371/journal.pone.0029198

**Published:** 2012-02-22

**Authors:** Tine Glendorf, Louise Knudsen, Carsten E. Stidsen, Bo F. Hansen, Anne Charlotte Hegelund, Anders R. Sørensen, Erica Nishimura, Thomas Kjeldsen

**Affiliations:** 1 Diabetes Research Unit, Novo Nordisk A/S, Maaloev, Denmark; 2 Diabetes Research Unit, Novo Nordisk A/S, Gentofte, Denmark; Omaha Veterans Affairs Medical Center, United States of America

## Abstract

**Background:**

Insulin analogues comprising acidic amino acid substitutions at position B10 have previously been shown to display increased mitogenic potencies compared to human insulin and the underlying molecular mechanisms have been subject to much scrutiny and debate. However, B10 is still an attractive position for amino acid substitutions given its important role in hexamer formation. The aim of this study was to investigate the relationships between the receptor binding properties as well as the metabolic and mitogenic potencies of a series of insulin analogues with different amino acid substitutions at position B10 and to identify a B10-substituted insulin analogue without an increased mitogenic to metabolic potency ratio.

**Methodology/Principal Findings:**

A panel of ten singly-substituted B10 insulin analogues with different amino acid side chain characteristics were prepared and insulin receptor (both isoforms) and IGF-I receptor binding affinities using purified receptors, insulin receptor dissociation rates using BHK cells over-expressing the human insulin receptor, metabolic potencies by lipogenesis in isolated rat adipocytes, and mitogenic potencies using two different cell types predominantly expressing either the insulin or the IGF-I receptor were systematically investigated. Only analogues B10D and B10E with significantly increased insulin and IGF-I receptor affinities as well as decreased insulin receptor dissociation rates displayed enhanced mitogenic potencies in both cell types employed. For the remaining analogues with less pronounced changes in receptor affinities and insulin receptor dissociation rates, no apparent correlation between insulin receptor occupancy time and mitogenicity was observed.

**Conclusions/Significance:**

Several B10-substituted insulin analogues devoid of disproportionate increases in mitogenic compared to metabolic potencies were identified. In the present study, receptor binding affinity rather than insulin receptor off-rate appears to be the major determinant of both metabolic and mitogenic potency. Our results also suggest that the increased mitogenic potency is attributable to both insulin and IGF-I receptor activation.

## Introduction

In order to improve insulin therapy for diabetic patients, injectable insulin analogues with different pharmacokinetic profiles have been developed to mimic the physiological plasma insulin profiles of endogenously produced insulin. In the β-cell, HisB10 plays an important role in hexamer formation by the coordination of zinc [1] and functions in processing and trafficking of insulin through the secretory pathway [2]. An inverse relationship was discovered between subcutaneous absorption and insulin self-association, and this particular property of HisB10 guided the design of the fast-acting X10 analogue (also known as AspB10) [3]. Insulin analogues displaying a reduced propensity towards self-association were anticipated to act more rapidly than regular human insulin and this was in fact demonstrated for insulin X10 [3], [4].

Insulin and IGF-I are two closely related proteins that share similarities in both primary and tertiary structure [5]–[7]. The homology is paralleled by similarities in the structures of their receptors, the insulin receptor (IR) and the IGF-I receptor (IGF-IR) [8]–[10]. Insulin and IGF-I share a common overlapping binding site on the two receptors, which comprises structural differences in the regions governing ligand specificity [9], [11]. Insulin and IGF-I therefore bind with high affinity to their cognate receptors, but are also able to bind with low affinity to the non-cognate receptor [7], [12]. A commonly held view is that insulin primarily induces metabolic effects, whereas IGF-I is thought to regulate more mitogenic processes [13], [14]. However, in the proper cellular context, insulin can induce a mitogenic response through the IR and inversely, IGF-I can exert insulin-like metabolic effets through the IGF-IR [15], [16].

Certain insulin analogues including insulin X10, have been found to display disproportionately enhanced mitogenic compared to metabolic potencies [17]–[19]. Compared to human insulin, insulin X10 displays a 2-fold increase in metabolic potency, but a significantly higher increase in the mitogenic activity compared to human insulin of 3–20 fold depending on the cell type being employed [17], [18], [20]–[22]. Unfortunately, insulin X10 also showed an increased carcinogenic effect in female rats [23], and it has therefore been tempting to speculate that this effect was associated with the increased mitogenic potency; however, this has not been proven and the precise molecular basis underlying the increased tumorogenic potential is still unknown. Insulin X10 also has an increased affinity for both the IR and the IGF-IR [17], [19], [21], [24] and a slower IR off-rate [18], [19], [24], but whether the increased mitogenicity relates to the increased affinity for the IGF-IR, the time of occupancy of the IR, or a combination of both IR and IGF-IR-mediated effects has been much debated [25]–[27] and remains to be fully elucidated.

Given the previous history with insulin X10, but taking into account the distinctive properties attained by HisB10 replacement, we wanted to examine the relationships between the receptor binding properties as well as the metabolic and mitogenic potencies of the analogues with different amino acid substitutions at position B10 in order to explore the possibility of identifying a B10-substituted insulin analogue without an increased mitogenic to metabolic potency ratio seen for insulin X10. In this study, several B10-substituted insulin analogues devoid of disproportionate increases in mitogenic compared to metabolic potencies were identified; of which B10V resembled human insulin the most. Only analogues B10D and B10E with significantly increased IR and IGF-IR affinities as well as decreased IR dissociation rates displayed enhanced mitogenic potencies in both cell types employed for mitogenicity determination.

## Materials and Methods

### Materials

Human insulin, [^125^I]-TyrA14-labelled insulin, human IGF-I, [^125^I]-Tyr31-labelled IGF-I, and immobilized *Achromobacter lyticus* (ALP) protease were from Novo Nordisk A/S. Receptor binding assays were performed using solubilized human IR and human IGF-IR semipurified by wheat germ agglutinin chromatography (according to the method from [28]) from baby hamster kidney (BHK) cells, which were stably transfected with the pZem vector containing either the human IR isoform A (IR-A), IR isoform B (IR-B) or IGF-IR insert (as recently described by [29]). Rats (Wistar, male, 150–200g) were acquired from an authorized breeding company (Taconic Europe A/S, Lille Skensved, Denmark) and housed according to standard procedures until sacrifice. The experiments with the primary rat adipocytes were performed according to the Animal Experiments Inspectorate (The Danish National Authority, proclamation no. 1273, December 8^th^ 2008 based on the animals rights law no. 1343 of December 12^th^ 2007), where tissue sampling from sacrificed animals does not require a permit. The IR-specific antibody 83-7 [30], IGF-IR specific antibody 24–31 [31] were licensed from Dr. K. Siddle (University of Cambridge, Cambridge, UK). Other chemicals used were of analytical grade or higher from Sigma-Aldrich.

### Cells

BHK cells overexpressing the A-isoform of the human insulin receptor (BHK-hIR) were prepared essentially as described in [32] (parent cell line BHK tk-ts13, cat. no. CRL-1632, ATCC). The L6 rat muscle myoblast cell line stably transfected with the human IR-A (L6-hIR) were the same as recently described [33] (parent cell line L6, cat. no. CRL-1458, ATCC). Clonetics® Human mammary epithelial cells (HMECs) were obtained from Lonza, Walkersville Inc., USA (cat. no. CC2251).

### Analogue expression, purification and labelling

Vector construction, precursor expression, conversion by ALP, quantification, and purification of the insulin analogues were performed as previously described (in [34] and references therein). In brief, insulin precursor DNA constructs were transformed into *Saccharomyces cerevisiae* (strain MT663) and expressed as proinsulin-like fusion proteins with a removable N-terminal spacer peptide and a mini C-peptide. The secreted analogue precursors were captured from cell-free acidified culture supernatant on a cation exchange column. The eluted precursors were enzymatically converted into mature two-chain desB30 insulin analogues (lacking the amino acid at position B30) by ALP treatment and further purified by preparative reverse-phase high-performance liquid chromatography, from which the main protein peak was collected and lyophilized. Full conversion of the resulting desB30 analogues were verified by matrix-assisted laser desorption ionization time-of-flight mass spectrometry. Analogue concentrations and purity were determined by reverse-phase high-performance liquid chromatography. [^125^I]-TyrA14-labelled analogues were prepared using the lactoperoxidase method as previously published [35].

### Binding assays

IR competition binding assays were performed on both isoforms of the receptor in a scintillation proximity assay as recently published [34]. IGF-IR competition binding assays were conducted essentially as the IR binding assays in 96-well plates (OptiPlate-96, PerkinElmer Life Sciences) on an Eppendorf epMotion 5075 robot. Assays were initiated by making dilution series (nine dilutions, 5-fold each) in binding buffer of a purified human insulin standard (starting from 7.5 µM) and a purified insulin analogue (starting from 7.5 µM) or IGF-I (starting from 0.1 µM) (n = 4 on each plate for the human insulin standard, insulin analogue or IGF-I) followed by addition of [^125^I]-labelled IGF-I, anti-IGF-IR mouse antibody (24–31), solubilized human IGF-IR, and scintillation proximity assay beads (SPA PVT Antibody-Binding Beads, Anti-Mouse Reagent, GE Healthcare) resuspended in binding buffer. The solubilized receptors were used at a suitable concentration, adjusted to give <20% bound tracer when no competing ligand was added to avoid ligand depletion. The buffer consisted of 100 mM HEPES (pH 7.8), 100 mM NaCl, 10 mM MgSO_4_, and 0.025% (v/v) Tween-20. Plates were incubated with gentle shaking for 24 h at 22°C, centrifuged, and counted in a TopCount NXT (PerkinElmer Life Sciences). IC_50_ values were determined using the four parameter logistic model [36] assuming constant slope, basal and maximal response. The IR affinities (picomolar affinity range) and the IGF-IR affinities (nanomolar affinity range) of the insulin analogues were calculated relative to that of the human insulin standard [IC_50(human insulin)_/IC_50(analogue)_×100%] measured within the same plate.

### Metabolic potency determination

Metabolic potencies were determined by lipogenesis (rFFC assay) using isolated primary rat adipocytes essentially as described in [37], [38]. Briefly, the epididymal fat pads were removed from killed Wistar rats and placed in degradation buffer (Krebs buffer, 1% HSA, 4 mg/mL collagenase (Worthington), and 2 mg/mL glucose) under vigorous shaking for 1 h at 37°C. The cell suspension was filtered, washed twice, and resuspended in incubation buffer (Krebs buffer, 1% HSA). Aliqouts of 100 µL were incubated for 2 h with gentle shaking at 37°C in 96-well PicoPlates (PerkinElmer Life Sciences) with 10 µL of glucose solution containing 12.5 µL/mL D-[3-^3^H]-glucose (GE Healthcare) and 0.08 mg/mL glucose together with 10 µL of increasing concentrations of human insulin or insulin analogue. The incubation was stopped by the addition of 150 µL MicroScint-E (Packard) and plates were counted in a TopCount NXT. EC_50_ values (picomolar range) were determined using the four parameter logistic model [36] and the metabolic potencies of the insulin analogues were calculated relative to that of the human insulin standard [EC_50(human insulin)_/EC_50(analogue)_×100%] measured within the same plate.

### Mitogenic potency determination

HMECs were maintained according to the manufactures recommendations in mammary epithelial growth media (MEGM®) containing SingleQuots® of supplements and growth factors (bovine pituitary extract, hydrocortisone, epidermal growth factor, insulin and gentamicin/amphotericin-B) (all from Cambrex). For experiments, cells were seeded at a density of 4×10^3^ cells/well in 96-well plates and incubated for 24 h in assay medium (MEGM® except insulin) after which increasing concentrations of ligand were added. Plates were incubated for 72 h at which 0.125 μCi/well [^3^H]-thymidine (Amersham Biosciences) was added at t = 70 h. Cells were harvested using a cell harvester and scintillation liquid added to the dried filter plates after which radioactivity was counted in a TopCount NXT (all from PerkinElmer Life Sciences). Potencies were calculated relative to that of the human insulin standard [EC_50(human insulin)_/EC_50(analogue)_×100%] measured within the same plate. The dose-response curves were fitted by non-linear regression using GraphPad Prism 4 (GraphPad Software, Inc).

L6-hIR cells were cultured at 37°C in a 5% CO_2_ humidified atmosphere in Nunc culture flasks with growth medium consisting of Dulbecco's Modified Eagle Medium (DMEM) supplemented with 10% (v/v) FBS, 1% (v/v) Pen/Strep/Glu and 1 mg/ml G418 (all from Gibco). Cells were subcultured 3 times per week with a split ratio of 1∶10 for two days and 1∶20 for three days. DNA synthesis was quantified as [^3^H]-thymidine incorporation into genomic DNA according to an optimized method by C. Bonnesen and M. B. Oleksiewicz (personal communication). Briefly, the rat L6 myoblasts overexpressing the A-isoform of the human insulin receptor were synchronized by topoinhibition and serum starvation and stimulated for 18–19 hours in medium containing 0.1% serum supplemented with increasing concentrations of ligand. The cells were pulse-labelled with 0.125 μCi/well of [^3^H]-thymidine for 2 h and transferred to filter plates using a cell harvester. Subsequently, 30 µL/well of Microscient O scintillation liquid was added to the dried filter plates and radioactivity measured in a TopCount NXT (all from PerkinElmer Life Sciences). The mitogenic potencies were calculated relative to that of the human insulin standard [EC_50(human insulin)_/EC_50(analogue)_×100%] measured within the same plate by non-linear regression using GraphPad Prism 5 (GraphPad Software, Inc).

### Determination of insulin receptor dissociation rate constants

BHK-hIR cells were cultured at 37°C in a 5% CO_2_ humidified atmosphere in Nuncleon culture flasks with growth medium consisting of DMEM supplemented with 10% (v/v) FBS, 100 µg/ml streptomycin, 100 U/ml penicillin (all from Cambrex), and 1 µmol/L methotrexate (Lonza). Cells were subcultured at a 1∶25 split ratio every 3–4 days for routine maintenance. For experiments, cells were seeded on 24-well Nunclon plates coated with Poly-L-lysine at a density of 8×10^3^ cells/well (adjusted to give 5–10% bound tracer in the absence of unlabelled ligand) in growth medium. Cells were used at ∼80% confluence and were incubated with [^125^I]-labelled human insulin or insulin analogue (50 pM final concentration) for 2.5 h at 4°C. Cells were washed quickly with ice-cold assay buffer consisting of DMEM with 0.1% (v/v) γ-globulin and Complete™ protease inhibibitor mixture (Roche Diagnostics). The dissociation of radioactively labelled ligand was measured at different time points after the addition of assay buffer containing 1 µM unlabelled human insulin at 4°C. The receptor-bound tracer was counted on a Cobra II γ-counter (PerkinElmer Life Sciences) and the dissociation rate constants were calculated using GraphPad Prism 4 (GraphPad Software, Inc).

## Results and Discussion

A series of ten different insulin analogues each comprising a single amino acid substitution at position B10 were systematically evaluated with respect to IR-A, IR-B, and IGF-IR binding, IR off-rates as well as metabolic and mitogenic potencies compared to human insulin. The B10H analogue (desB30 human insulin) was included in all the assays, thus serving as an internal control. The analogues tested were B10A, B10R, B10D, B10Q, B10E, B10H, B10I, B10F, B10W, and B10V, which represent a diverse panel of insulin analogues with different amino acid side chain characteristics at position B10.

### Insulin receptor binding

**Figure 1:**
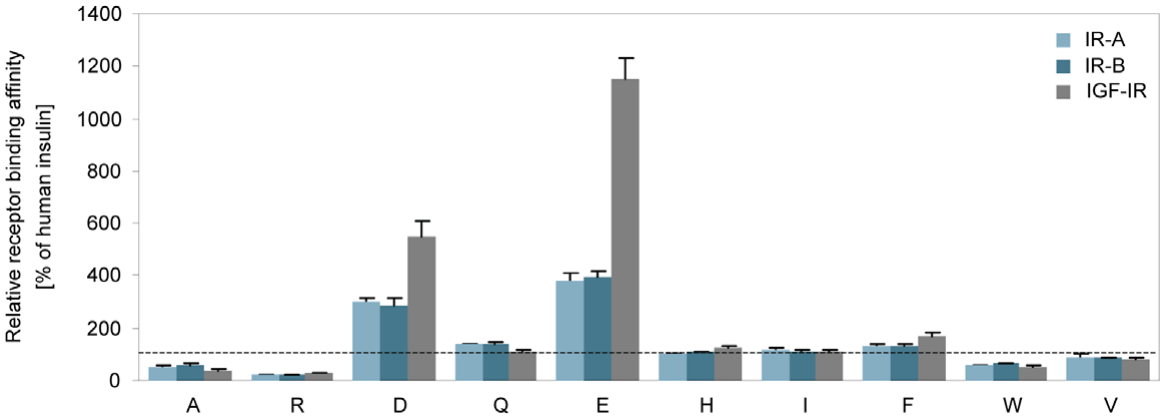
Relative receptor binding affinities. Receptor binding affinities for the solubilised IR-A (light teal), IR-B (dark teal) and IGF-IR (gray) relative to human insulin. The gray dotted line represents 100% binding affinity compared to that of human insulin. Data are means ± SD (n = 3).

The relative insulin receptor binding affinities of the analogues were determined using human insulin receptors (full-length) purified from BHK cells (see Table 1 and Figure 1). For each analogue, receptor binding affinities were determined on both the A and B isoform of the IR. All the analogues displayed a balanced IR-A/IR-B affinity ratio (R^2^ = 0.99, p<0.0001 between the relative IR-A and IR-B binding affinities). As previously determined [34], [39], [40], the negatively charged Asp and Glu substitutions (analogues B10D and B10E) caused 3-4-fold increases in IR affinity, while analogues B10Q, B10I, and B10F exhibited only moderately enhanced IR binding affinities. The most pronounced decrease in receptor binding (5-fold) was observed for the B10R analogue, whereas analogues B10A and B10W displayed 2-fold reductions in IR binding affinity. Analogue B10V had a relative IR affinity close to that of human insulin.

**Table 1:** Relative receptor affinities, metabolic and mitogenic potencies, and IR off-rates [% of human insulin].

	Receptor binding									Lipogenesis			Mitogenic potency								
Analogue	IR-A			IR-B			IGF-IR			rFFC			HMEC			L6-hIR			IR off-rate		
B10A	51	±	5	58	±	5	38	±	3	74	±	12	25	±	7	43	±	8	86	±	2
B10R	21	±	2	24	±	2	28	±	2	46	±	6	37	±	16	18	±	2	149	±	7
B10D	297	±	21	285	±	31	548	±	59	215	±	22	568	±	165	221	±	43	20	±	0.3
B10Q	137	±	5	139	±	10	109	±	7	103	±	20	180	±	54	101	±	18	83	±	2
B10E	382	±	30	399	±	22	1151	±	77	226	±	23	888	±	257	257	±	127	14	±	2
B10H	105	±	1	107	±	3	122	±	8	98	±	10	86	±	25	92	±	21	101	±	6
B10I	118	±	6	113	±	6	113	±	2	61	±	11	69	±	19	38	±	5	68	±	3
B10F	135	±	6	134	±	4	167	±	15	52	±	3	90	±	14	48	±	3	54	±	1
B10W	60	±	2	65	±	4	53	±	2	27	±	10	39	±	12	38	±	1	46	±	1
B10V	90	±	10	85	±	1	84	±	6	67	±	13	65	±	30	97	±	24	93	±	6

All assays were performed in at least three independent experiments. Data are means ± SD and presented relative to human insulin. For human insulin, IR assay IC_50_ values were in the picomolar affinity range, IGF-IR assay IC_50_ values were in the nanomolar affinity range, rFFC assay EC_50_ values were in the picomolar range, and mitogenic assay EC_50_ values were in the nanomolar range (HMECs) and low nanomolar range (L6-hIR). The dissociation rate constant for human insulin was (3.7±0.3×10^−2^ min^−1^).

### IGF-I receptor binding

The relative IGF-I receptor binding affinities of the analogues were determined using human IGF-I receptors (full-length) purified from BHK cells. As can be seen from Table 1 and Figure 1, the IGF-IR was more affected by the amino acid substitutions than the IR and the relative affinities ranged from 28% for B10R to 1151% for B10E. Compared to IR binding, analogue B10E had a 3-fold higher relative IGF-IR binding affinity (L. Schäffer [41] determined the difference to be 2-fold), which in agreement with the fact that the amino acid residue corresponding to HisB10 in insulin is a Glu residue in the homologues IGF-I molecule, a residue recently identified to be very important in IGF-IR binding [42].

Interestingly, the internal desB30 human insulin control (B10H) displayed a slight increase in relative binding affinity compared to human insulin, indicating that the basic C-terminal LysB29 residue has an impact on IGF-IR binding as opposed to IR binding, which is unaffected by the removal of ThrB30. This phenomenon was also observed in a previous study [20], in which B10D, desB30 was found to have a higher affinity for the IGF-IR than the corresponding full-length B10D analogue (insulin X10). In this study, the B10D analogue displayed a 5-fold increase in IGF-IR binding compared to human insulin, which is in good agreement with earlier results [20]. Analogues with decreases in IR binding affinities compared to human insulin also had decreased IGF-IR binding affinities and ranked in the same order on both receptor types. Analogue B10F displayed a small increase in IGF-IR compared to IR binding as with analogue B10H, whereas the opposite was observed for the B10Q analogue. The B10V analogue displayed a balanced IR to IGF-IR binding affinity ratio and had a relative affinity close to that of human insulin.

### Metabolic potency

The metabolic potencies of the analogues were determined by measuring the dose-dependent stimulation of lipogenesis over a 2 h period in isolated rat adipocytes (see Table 1). The dose-response curves for the analogues and human insulin exhibited no significant differences in minimal and maximal responses (∼12-fold stimulation) or in the slopes. The metabolic potencies ranged from 27% for B10W to 226% for B10E relative to that of human insulin. As observed in both the IR and IGF-IR binding experiments, analogues B10D and B10E were the most potent analogues in this assay and the metabolic potencies determined in this study are in good agreement with previous findings [18].

Compared to the relative IR binding potencies, analogues B10A and B10R were more metabolically potent; whereas the metabolic potencies of analogues B10I, B10F, and B10W were 2–2.5-fold lower than their relative IR binding affinities. Analogues B10Q and B10V only displayed slight decreases in relative metabolic potency compared to IR binding. It has previously been shown for several different insulin analogues that their metabolic potencies correlated well with their equilibrium IR binding affinities [17], [19]; however, in the present study, the majority of the analogues (including B10D and B10E) displayed slightly decreased relative metabolic potencies compared to relative IR binding potencies and a less pronounced degree of correlation (R^2^ = 0.87, p<0.0001) between IR binding and metabolic potency of the B10 analogues was observed. As with IR binding, the internal desB30 insulin control displayed the same relative potency as human insulin.

### Mitogenic potency

**Figure 2:**
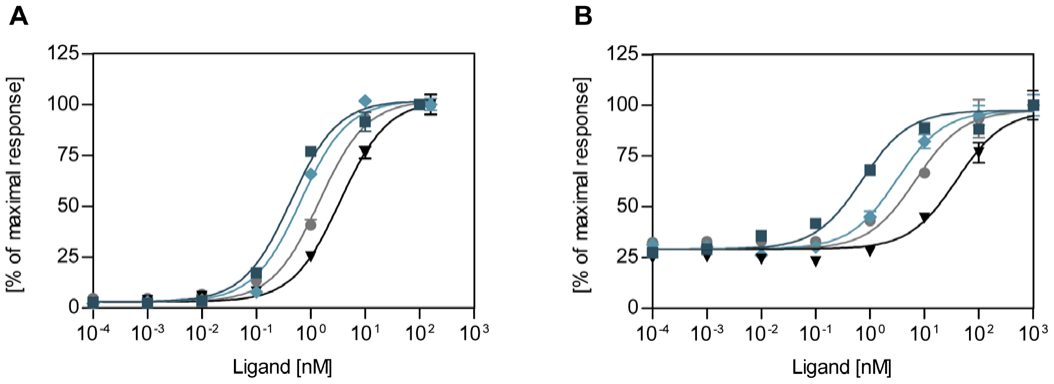
Representative dose-response profiles for mitogenic potency determination. Human insulin (•) or insulin analogue (B10D (♦), B10E (▪), or B10A (▾)) stimulated incorporation of [^3^H]-thymidine into DNA is shown in (**A**) L6-hIR cells and (**B**) HMECs. Data points are means ± SEM (n = 3).

To assess the mitogenic potencies of the analogues, two different cell types predominantly expressing either the IR or the IGF-IR, were employed in order to detect the mitogenic effects of the analogues mediated mainly through either of the two receptor types. Cell growth was determined by [^3^H]-thymidine incorporation into DNA in the rat L6 myoblast cell line overexpressing the human IR-A (L6-hIR) as well as in primary, non-transformed human mammary epithelial cells (HMECs), which express approximately 20-fold more IGF-I receptors than insulin receptors [20]. In both assays, no significant differences in minimal and maximal responses or in the slopes of the dose-response curves were observed between human insulin and the insulin analogues (see Figure 2). In the HMECs, maximal stimulation caused a 3–6-fold increase in growth response over basal (EC_50_ values were in the nM range), while ∼20-fold responses were observed in the L6-hIR cells (EC_50_ values were in the low nM range). The absolute mitogenic potency of human insulin was therefore lower in the HMECs compared to L6-hIR cells as expected if the effect was mediated by the IGF-IR in the HMECs, but through the IR in the L6-hIR cells. The relative mitogenic potencies of the analogues compared to human insulin are presented in Table 1.

The broader range of receptor binding affinities determined on the IGF-IR compared to the IR was paralleled in the mitogenic potencies of the analogues, where potencies ranged from 18% to 257% in the L6-hIR cells and from 25% to 888% in the HMECs. In general, the relative mitogenic potencies determined in HMECs correlated (R^2^ = 0.96, p<0.0001) with the relative IGF-IR binding affinities of the analogues, while a correlation (R^2^ = 0.90, p<0.0001), though less pronounced, was observed between the relative IR binding affinities and the relative mitogenic potencies determined in the L6-hIR cells. This is in good agreement with previous reports [17], [20], [43] in which analogues with increased IGF-IR binding affinities were found to induce enhanced mitogenic responses in cells expressing a high proportion of IGF-I receptors compared to insulin receptors.

Only analogues B10D and B10E were more potent than human insulin in stimulating cell growth in both cell systems. Compared to human insulin, B10D and B10E displayed 2- and 3-fold increases in relative mitogenic potencies in the L6-hIR cells, while 6-fold and 9-fold increases were observed in the HMECs, respectively. Analogue B10Q, which exhibits the highest degree of structural resemblance to B10E, displayed almost a 2-fold increase in mitogenic potency in the HMECs, while the remainder of the analogues were less mitogenic than human insulin in both of the cell types employed. The correlation between IGF-IR binding and mitogenicity measured in the HMECs as well as the correlation between IR binding and mitogenic potency measured in L6-hIR cells were therefore also less obvious, when looking at the analogues with relatively small changes in IGF-IR binding affinity compared to human insulin. Analogue B10H displayed relative mitogenic potencies comparable to that of human insulin in both cell types employed even though its IGF-IR binding affinity was slightly higher than that of human insulin.

In general, the mitogenic potencies determined in L6-hIR cells correlated well (R^2^ = 0.95, p<0.0001) with the metabolic potencies of the analogues. This was also valid for analogues B10D and B10E, which were equally potent in both the rFFC and the L6-hIR assay. However, when comparing the metabolic potencies with the mitogenic potencies of the analogues determined in HMECs, no clear correlation was identified. Analogues B10D and B10E with highly increased receptor affinities, showed 3–4-fold increases in the mitogenic (HMEC) to metabolic potency ratio, respectively. Taking the distribution of insulin and IGF-I receptors as well as the correlations between mitogenicity and receptor binding into account, it seems that the mitogenic response measured in the HMECs predominantly was mediated through the IGF-I receptor, whereas the mitogenic potency of the analogues in the L6-hIR cells mainly was mediated via the insulin receptor.

### Insulin receptor dissociation rates

**Figure 3:**
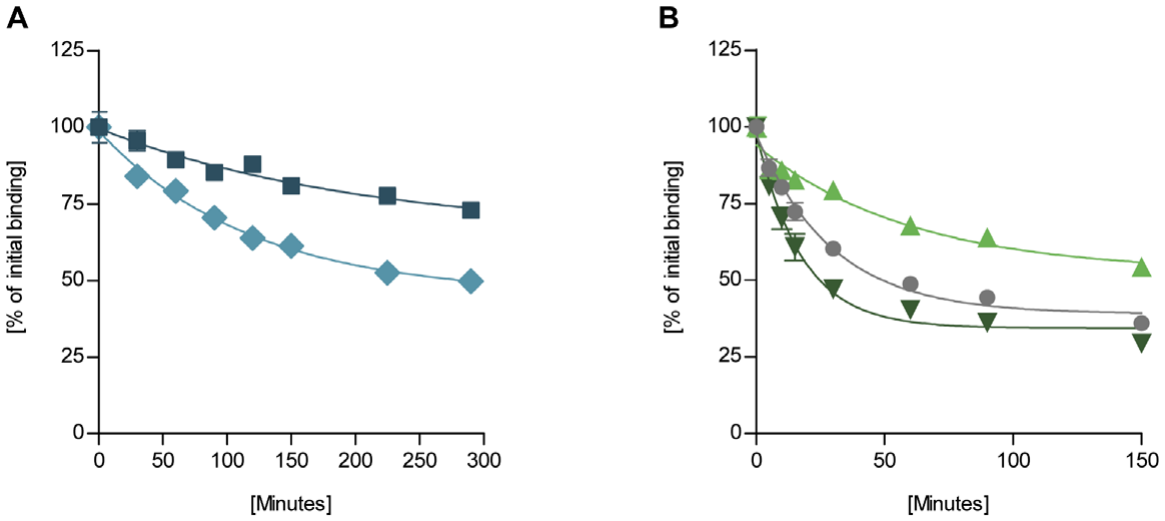
Representative dissociation curves of [^125^I]-labelled insulin or analogue from BHK-hIR cells. Dissociation was measured at different time points and the residual binding expressed as a percentage of initial binding. Dissociation of (**A**) B10D (♦) and B10E (▪); (**B**) B10W (▴), human insulin (•), and B10R (▾). Data points are means ± SEM (n = 3).

The dissociation rate constants from the insulin receptor were determined using BHK cells overexpressing the human IR-A. Dissociation of [^125^I]-labelled human insulin or insulin analogue was determined as a function of time in the presence of an excess of unlabelled insulin. Representative dissociation time courses are presented in Figure 3 and the results and the dissociation rate constants relative to human insulin (3.7±0.3×10^−2^ min^−1^) are listed in Table 1. Dissociation was measured over a period of 2.5 h; however, analogues B10D and B10E dissociated 5- and 7-fold slower from the receptor, respectively, compared to human insulin and dissociation was therefore measured over a 5 h period for these two analogues in order to get a better curve fit and thereby a more precise determination of the dissociation rate constant.

Analogues B10Q, B10I, and B10F, which all displayed slight increases in IR binding affinity, also displayed a slower dissociation from the IR than human insulin. Analogue B10W clearly had a more flat dissociation curve than human insulin and exhibited a 2-fold decrease in IR dissociation rate even though it displayed a relative IR binding affinity lower than human insulin. However, the relative IR affinity was determined by equilibrium binding and the decrease in off-rate may therefore be paralleled by a decrease in on-rate, but association rates were not determined in this study. Analogues B10A and B10R displayed 2-fold and 5-fold reductions in relative IR binding affinity, respectively, but whereas B10R displayed a 1.5-fold increase in IR off-rate, analogue B10A exhibited a minor decrease in off-rate. This may again be counterbalanced by a decrease in the IR on-rate in order to achieve a 2-fold reduction in IR binding affinity, but it may also be attributable to an underestimation of the accelerated dissociation [44], which is most evident in the initial phase of the dissociation curve.

It has been observed in previous studies that insulin analogues with increased mitogenic potencies dissociated very slowly from the IR. The sustained activation of the IR was found to correlate with increased mitogenicity, suggesting that the enhanced mitogenic response was attributable to an increased duration of the insulin signal at the receptor level [14], 18,19. In the present study, analogues B10D and B10E displayed very low IR dissociation rates of 20% and 14%, respectively, relative to human insulin, which was paralleled by similar increases in the mitogenic potencies when measured in the HMECs. The mitogenic (HMEC) to metabolic potency ratio of these slowly dissociating analogues was increased by 3 fold for B10D and 4 fold for B10E. In the L6-hIR cells on the other hand, the 5- and 7-fold decreases in IR off-rate exceeded that of the approximate 2-fold increases in mitogenic potency. While analogues B10D and B10E displayed the lowest IR-off rates and highest mitogenic potencies, the correlation between receptor dissociation rate and mitogenicity was less clear for the remaining B10 analogues. The aromatically substituted B10F and B10W analogues both exhibited 2-fold decreases in IR off-rate, but were less mitogenic than human insulin in the two cell types employed. The dissociation rates of analogues B10H and B10V were similar to that of human insulin.

### Concluding remarks

It would be of great interest to fully elucidate the molecular mechanisms underlying the enhanced mitogenicity observed for certain insulin analogues. However, the analysis of the metabolic and mitogenic effects elicited by the IR and IGF-IR both *in vitro* and *in vivo* is complicated. Factors complexifying clarification include co-expression of the receptors on the cell surface of most cells and the formation of hybrids [45]–[47], the large homology and structural resemblance of insulin and IGF-I as well as their receptors leading to non-cognate receptor binding [7], [8], [10], and the overlapping signalling pathways involving phosphorylation of the same intracellular receptor substrates and downstream signalling molecules [13], [48]. In addition, insulin analogues may also exhibit differences in their susceptibility to degradation and cellular processing [49], [50] that may influence their metabolic as well as mitogenic potencies thus complicating comparison of the biological properties of different insulin analogues in order to deduce the molecular characteristics affecting the metabolic/mitogenic potency ratio.

Recent reports have also suggested that the IR isoform expression pattern [47], [51], the IR/IGF-IR ratio expressed on the cell surface [25] as well as internalization and signalling from different cellular compartments play a role in determining the mitogenicity of insulin analogues [52]–[54]. The metabolic and mitogenic properties of insulin analogues may therefore result from a complex combination of the expression levels of signalling molecules (e.g. IRSs and Shcs) as well as the different events taking place upon and after receptor binding and are therefore not fixed inherent characteristics, but depend on cell type and the specific biological end-point being studied.

Previous studies investigating the mitogenicity of insulin analogues with amino acid substitutions at position B10 have only included analogues with acidic Asp or Glu substitutions, but in this study, a panel of insulin analogues with amino acids comprising different side chain characteristics were systematically examined. Even though analogues B10D and B10E displayed increased mitogenic potencies and dissociated very slowly from the IR, no apparent correlation between insulin receptor occupancy time and mitogenicity was observed for the remaining B10-substituted insulin analogues with less pronounced changes in receptor affinities and IR dissociation rate constants. Instead, the presented data suggest that receptor binding affinity rather than insulin receptor off-rate seem to be the main predictor of both metabolic and mitogenic potency.

Together, our results suggest that the enhanced mitogenicity is attributable to both IR and IGF-IR activation, which is in good agreement with the fact that the two receptors share a common binding site. However, we also show that minor increases in receptor binding alone do not entail increased mitogenicity. Only the acidic Asp and Glu substitutions, which give rise to highly increased IR and IGF-IR binding affinities and at the same time, resemble the acidic substitution found at the corresponding position in IGF-I the most, entail significantly increased mitogenic potencies compared to human insulin in both cell types employed. This would suggest that future insulin analogues featuring ‘IGF-I-like’ substitutions causing significant increases in both IR and IGF-IR affinity and an enhanced mitogenic/metabolic potency ratio should be avoided.

In summary, several B10-substituted insulin analogues devoid of disproportionate increases in mitogenic compared to metabolic potencies were identified; of which analogue B10V taken as a whole resembled human insulin the most. Depending on the desired properties of future insulin analogues comprising a B10 substitution, the different physico-chemical properties of the amino acid side chain properties will have to be determined and taken into account.
